# Highly Efficient and Stable Removal of Arsenic by Live Cell Fabricated Magnetic Nanoparticles

**DOI:** 10.3390/ijms20143566

**Published:** 2019-07-21

**Authors:** Hyo Kyeong Kim, Sun-Wook Jeong, Jung Eun Yang, Yong Jun Choi

**Affiliations:** 1 School of Environmental Engineering, University of Seoul, Seoul 02504, Korea; 2 World Institute of Kimchi, Gwangju 61755, Korea

**Keywords:** *Deinococcus radiodurans* R1, bioremediation, magnetic nanoparticle, arsenic, adsorption

## Abstract

As concerns about public health and environmental problems regarding contamination by toxic substances increase worldwide, the development of a highly effective and specific treatment method is imperative. Although physicochemical arsenic treatment methods have been developed, microbial in vivo remediation processes using live cell fabricated nanoparticles have not yet been reported. Herein, we report the development of magnetic iron nanoparticles immobilized an extremophilic microorganism, *Deinococcus radiodurans* R1, capable of removing toxic arsenic species. First, in vivo synthesis of magnetic iron nanoparticles was successfully achieved with the *D. radiodurans* R1 strain and characterized by scanning electron microscopy-energy dispersive X-ray spectroscopy (SEM-EDX), dynamic light scattering (DLS), zeta-potential, Fourier transform infrared spectroscopy (FT-IR), X-ray diffraction (XRD), and X-ray photoelectron spectroscopy (XPS) analysis. Second, the maximum removal capacity of the magnetic iron nanoparticle-immobilized *D. radiodurans* R1 strain (DR-FeNPs) for arsenic [As(V)] was evaluated under the optimized conditions. Finally, the removal capacity of DR-FeNPs in the presence of various competitive anions was also investigated to simulate the practical application. More than 98% of As(V) was efficiently removed by DR-FeNPs within 1 h, and the removal efficiency was stably maintained for up to 32 h (98.97%). Furthermore, the possibility of recovery of DR-FeNPs after use was also suggested using magnets as a proof-of-concept.

## 1. Introduction

For several decades, contamination of aqueous environments and poisoning of drinking water by toxic substances has been a serious concern because of their adverse effects on public and human health [1]. Among the variety of toxic substances, arsenic is the most widespread chemical element that has the potential to endanger humanity by poisoning groundwater [2]. Low-level exposure to arsenic triggers dysfunctioning of the immune system, which leads to an increased risk of infection to lethal pathogens, such as H1N1 and swine flu viruses [3]. Moreover, long-term exposure to arsenic leads to significant mortality. Of special concern are chronic diseases such as skin cancer, lung cancer, and kidney cancers, which can also lead to postoperative complications [4]. Thus, the International Agency for Research on Cancer (IARC) designated arsenic compounds as group 1 carcinogens, and their provisional value recommended by the World Health Organization (WHO) and the United States Environmental Protection Agency (USEPA) has been adjusted to less than 10 μg/L [5,6].

To date, a wide variety of conventional physicochemical methods such as coagulation, precipitation, adsorption, membrane technologies, and electrodialysis have been developed for the removal of As(V) from the aqueous solution [7]. Despite its widespread use, some obstacles such as labor-intensity, high-cost, and secondary contamination have remained a challenge to be solved. Thus, much exploration of hybrid technologies to overcome those of hurdles is underway that goes beyond academic boundaries.

Nanoparticles with properties including strong adsorption capacity, high selectivity, and extensibility have been developed as an efficient and stable agent for the treatment of toxic substances in aqueous media. In particular, iron oxide magnetic nanoparticles (FeNPs) are attractive nanomaterials for the adsorption of arsenic contaminants because of their high affinity toward arsenic species. Chemically synthesized α-Fe_2_O_3_ nanoparticles exhibit about 95 mg/g and 47 mg/g of adsorption capacity toward As(III) and As(V), respectively, at a near neutral pH [8]. Another report stated that the maximum adsorption capacity of the biochar supported nanoscale zerovalent iron (nZVI/BC) is 124.5 g/kg at a pH of 4.1, in a batch study [9]. However, several drawbacks remain to be addressed for practical applications, such as the instability of nanomaterials under various environmental conditions, the energy-consuming synthesis method, and the recovery of unsettled adsorbents after the treatment [10,11,12].

Recently, bioremediation technology incorporating nanomaterials (Nano-biotechnology) has emerged as an alternative method to overcome the drawbacks of nanotechnology. For example, in vivo biosynthesis of novel metal nanoparticles such as Au, Ag, and Pd have been reported in recombinant *Escherichia coli* by increasing the cellular redox potential through overexpression of phytochelatin synthase (PCS) and metallothionein (MT) [13]. As another example, green synthesis of nanomaterials and the biosynthesis of zerovalent iron nanoparticles (ZVNI) have been reported using treatment with leaf extracts of *Eucalyptus globules* and 0.1 M FeSO_4_·7H_2_O for 1 min at room temperature [14]. In most cases, cellular metabolites with strong reducing power, such as carotenoids, flavonoids, and oxidative peptides, play an important role in the synthesis of biogenic nanoparticles. Thus, the extremophilic microorganisms containing strong antioxidants are widely used in the field of nanotechnology [15,16]. Among the extremophilic microorganisms, *Deinococcus radiodurans* R1 strain, a radiation-resistant bacteria, has great potential to be used in biogenic nanoparticles as it has evolved strong antioxidant systems and cellular metabolites [17]. Previously, we reported the development of a treatment method for radioactive iodine using gold nanoparticle-immobilized *Deinococcus radiodurans* R1 strain (*D. radiodurans* R1; DR). The gold nanoparticle-containing DR strain showed excellent removal capacity for radioactive iodine (>99%) in various aqueous solutions [18]. Moreover, the treatment method using a nanomaterial-embedded microorganism is superior to other previously reported remediation processes in terms of its efficiency and sustainability.

These observations led us to develop a rapid and highly efficient removal method for arsenic [As(V)] by adsorption using a magnetic iron nanoparticle-immobilized *D. radiodurans* R1 strain (DR-FeNPs). First, the magnetic nanoparticles were synthesized by DR strain and immobilized onto the cell. Next, adsorption conditions such as pH, contact time, amount of biomass, and initial concentration were evaluated for identifying the optimal conditions. In addition, the maximum removal efficiency, stability, and sustainability were also investigated under the optimized conditions. Finally, the practical application of DR-FeNPs for As(V) removal was verified under the presence of competitive anions and the recovery of used DR-FeNPs using magnets was also investigated as a proof-of-concept.

## 2. Results

### 2.1. Establishment of Live Cell Fabricated Iron Nanoparticles (FeNPs)

**Figure 1:**
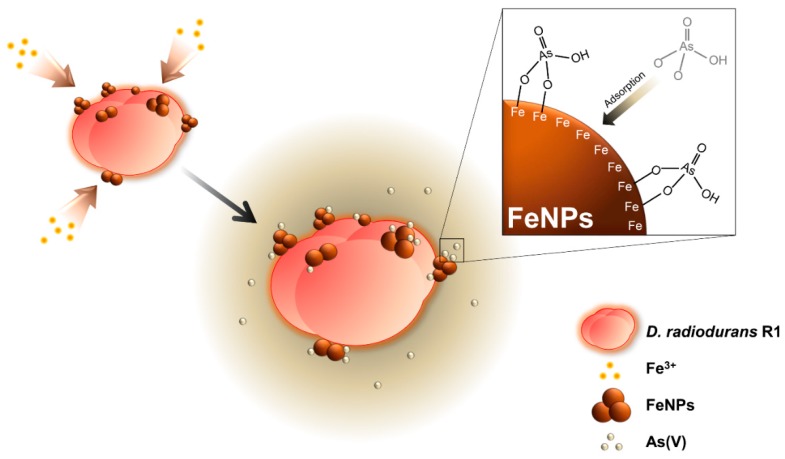
Schematic illustration of an overall strategy for the adsorption of As(V) using iron oxide magnetic nanoparticles (FeNPs) immobilized *D. radiodurans* R1.

**Figure 2:**
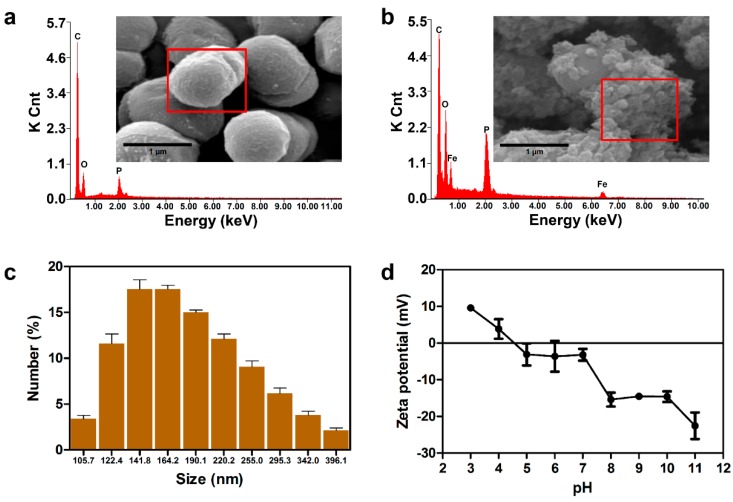
Scanning electron microscopy-energy dispersive X-ray spectroscopy (SEM-EDX) analysis of (**a**) *D. radiodurans* R1 and (**b**) iron nanoparticle immobilized *D. radiodurans* R1 (DR-FeNPs). Inset represents 50 kX magnified SEM images of *D. radiodurans* R1 without (left) or with (right) FeCl_3_; (**c**) dynamic light scattering (DLS) analysis of FeNPs synthesized by *D. radiodurans* R1 in vivo; (**d**) zeta potential analysis of DR-FeNPs. All experiments were performed in triplicate; error bars denote standard deviation.

*D. radiodurans* was selected as the host strain to make biogenic iron nanoparticles, as it expresses a variety of strong antioxidants and enzymes that provide reducing power, which is beneficial for the in vivo synthesis of nanomaterials [15]. The main strategy for the removal of arsenic [As(V)] using live cell fabricated iron nanoparticles is described in Figure 1. To synthesize iron nanoparticles using living bacterial cells, DR strain was cultured in TGY (Tryptone, Glucose, and Yeast) media supplemented with 6.25 mM of FeCl_3_. The color of the culture broth was found to gradually turn dark brown with increasing incubation time. To check whether the iron nanoparticles were synthesized, the nanomaterials were analyzed by scanning electron microscopy-energy dispersive X-ray spectroscopy (SEM-EDX) analysis. As shown in Figure 2a,b, the fairly irregular shape of iron nanoparticles (FeNPs) was efficiently synthesized in vivo and immobilized onto the DR strain (see the inset of each figure). Also, the peaks indicating Fe are displayed in the EDX spectrum compared to the same strain without supplementation of FeCl_3_. The dynamic light scattering (DLS) analysis also demonstrated that the size of FeNPs synthesized by the DR strain was about 141.8 nm to 164.2 nm (Figure 2c), which is relatively smaller than that of chemically synthesized FeNPs (192 ± 5.96 nm), which were also used for the removal of As(V) [19]. Previous studies on the characterization of nanomaterials reported that the adsorption capacity of the irregular and smaller size of nanomaterials tended to increase [20,21]. Thus, the adsorption capacity of FeNPs synthesized by DR strain was expected to be higher and more efficient compared with the chemically synthesized crystal iron nanoparticle. Furthermore, the isoelectric point (IEP) of between pH 4 to pH 5 (Figure 2d), which is similar to the IEP value of chemically synthesized Fe_2_O_3_ nanoparticle [22], was estimated by the zeta-potential analysis. On the basis of this IEP value, it can be predicted that the maximum removal efficiency of FeNPs for As(V) can be achieved at pH 4 or below, where the electrical charges of FeNPs (positive) and As(V) (negative) are different from each other [23]. Therefore, it was concluded that iron nanoparticles (FeNPs) were efficiently synthesized by the living DR strain and suitable for the removal of As(V) under the acidic condition [24].

### 2.2. Characterization of Morphological Parameters and Composition of FeNPs

**Figure 3:**
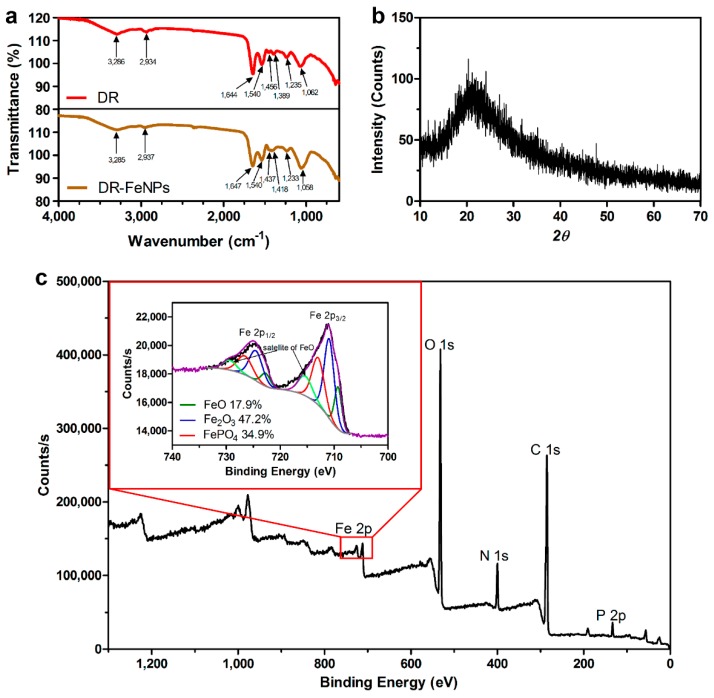
(**a**) FT-IR analysis of *D. radiodurans* R1 and iron nanoparticle immobilized *D. radiodurans* R1 (DR-FeNPs); (**b**) X-ray diffraction (XRD) pattern and (**c**) X-ray photoelectron spectroscopy (XPS) spectra of iron nanoparticle immobilized *D. radiodurans* R1 (DR-FeNPs). The red colored square indicated the magnified version of the Fe 2p signal.

Traditionally, because the functionality of nanomaterials is highly dependent on their morphology, they have been synthesized by chemical reactions and then modified to obtain the desired sizes and shapes. Unlike chemically synthesized nanomaterials whose shape and size are constant, biologically synthesized nanomaterials vary in size and shape. Thus, FT-IR, X-ray diffraction (XRD), and X-ray photoelectron spectroscopy (XPS) analysis were performed to characterize FeNPs synthesized by DR strain. First, the FT-IR analysis was performed to investigate the functional groups of biomolecules involved in the fabrication of FeNPs in vivo. As shown in Figure 3a, intensive band at 1644 cm^−1^, which corresponds to the stretching peaks of amide I group of polypeptides [15], was slightly shifted to 1647 cm^−1^. The bands at 1456 and 1389 cm^−1^ attributed the methylene scissoring vibration in the proteins and C–O stretching vibration from carboxylate ion were also shifted to 1437 and 1418 cm^−1^ during the process of in vivo synthesis of FeNPs [15,25,26,27]. Furthermore, the band at 1062 cm^−1^ representing P–O–C stretching vibrations was also shifted to 1058 cm^−1^ in FeNPs immobilized *D. radiodurans* R1 strain (DR-FeNPs), which is consistent with the result of EDX analysis composing the C, O, and P peaks, as described in Figure 2a,b. Thus, these results indicated that the amide and phospho groups might be involved in the formation of the live cell fabricated FeNPs.

Next, the XRD and XPS analyses were performed to determine the morphological parameters and composition of FeNPs. As shown in Figure 3b, the single broad diffused peak near the 20° in the 2*θ* in the range of 10°–70° was observed in the XRD analysis, indicating that the FeNPs synthesized by DR strain showed an amorphous structure, which is in coincidence with the previous study reporting the XRD pattern analysis for the characterization of FeNPs [28]. In the XPS analysis, the core-level spectra of C 1s, N 1s, O 1s, P 2p, and Fe 2p supported the functional groups involved in the synthesis of FeNPs, which is coincidence with FT-IR analysis. Furthermore, three types of FeNPs composed mainly of Fe_2_O_3_ (47.2%; 710.9 eV) and the rest of FeO (17.9%; 709.3 eV and 715.4 eV) and FePO_4_ (34.9%; 713.0 eV) were confirmed by the magnified spectrum of Fe 2p_2/3_ [29,30,31] (Figure 3c).

### 2.3. Removal of As(V) Using Iron Nanoparticle Immobilized D. radiodurans R1 (DR-FeNPs)

**Figure 4:**
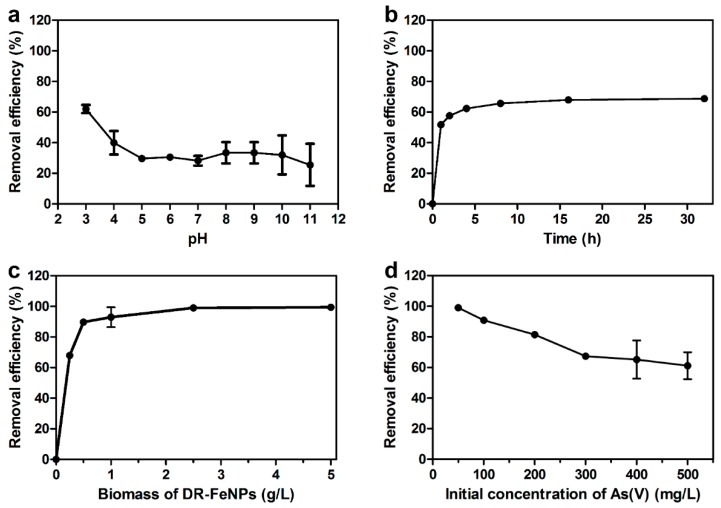
Optimization of adsorption conditions for maximum removal efficiency according to (**a**) pH, (**b**) contact time, (**c**) the amount of biomass, and (**d**) initial concentration of As(V). Experimental conditions were as follows: (**a**) contact time = 24 h, biomass = 0.2 g/L, initial concentration of As(V) = 50 mg/L; (**b**) initial pH = 3, biomass = 0.25 g/L, initial concentration of As(V) = 50 mg/L; (**c**) initial pH = 3, contact time = 16 h, initial concentration of As(V) = 50 mg/L; (**d**) initial pH = 3, contact time = 16 h, biomass = 2.5 g/L. All experiments were performed in triplicate; error bars denote standard deviation.

**Figure 5:**
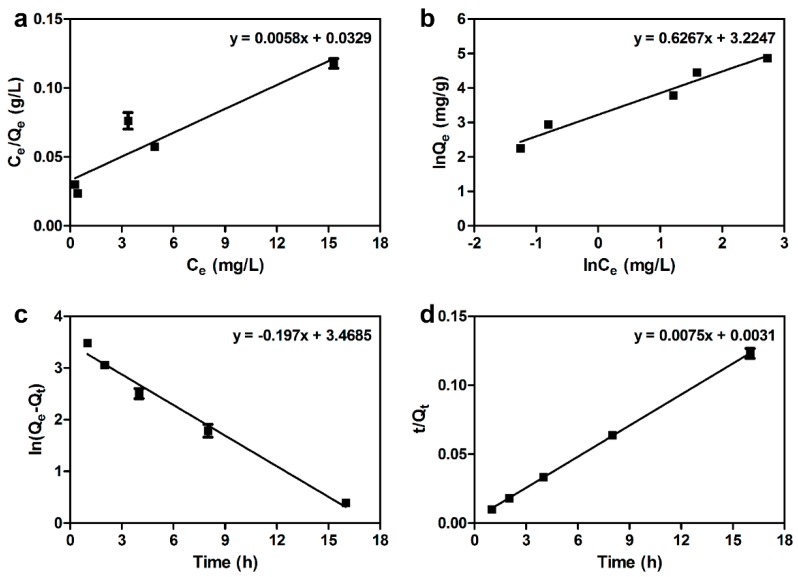
Adsorption isotherms plots of (**a**) Langmuir and (**b**) Freundlich; adsorption kinetics plots of (**c**) pseudo-first-order model and (**d**) pseudo-second-order model. All experiments were performed in triplicate; error bars denote standard deviation.

Next, the removal efficiency of As(V) using DR-FeNPs was tested in aqueous media. Before the investigation of the removal of As(V) by adsorption, the optimal pH, amount of biomass, contact time, and initial concentration of As(V), which are known to affect the adsorption capacity, were optimized under the various conditions. As shown in Figure 4, the maximum removal efficiency of the DR-FeNPs was observed at pH 3 (61.99% ± 2.61%), contact time 16 h (67.99% ± 1.99%), 2.5 g/L of biomass, and 50 mg/L of the initial concentration of As(V) (99.06% ± 0.15%). In addition, the Langmuir and Freundlich isotherm models were employed to understand the interaction between DR-FeNPs and As(V) during the process of adsorption [25]. The Langmuir isotherm model is represented by the following Equation (1).

(1) $$Q_{e}=\frac{Q_{0}K_{L}C_{e}}{1+K_{L}C_{e}},$$

where *Q_e_* (mg/g) is adsorption capacity at equilibrium, *Q*_0_ (mg/g) is maximum adsorption capacity, *C_e_* (mg/L) is the concentration of metal ions in aqueous solution at equilibrium, and *K_L_* (L/mg) is Langmuir adsorption constant. The Freundlich isotherm model is represented by the following Equation (2):

(2) $$Q_{e}=K_{F}C_{e}{}^{\frac{1}{n}},$$

where *K_F_* (L/g) and 1/*n* are Freundlich constant and adsorption intensity, respectively. The isothermal adsorption curve was fitted to the Freundlich model because the values of *R*^2^ for Freundlich model (*R*^2^ = 0.961) were higher than that of the Langmuir model (*R*^2^ = 0.870) (Table 1, Figure 5a,b). This result indicated that the removal of As(V) using DR-FeNPs could be explained by a heterogeneous surface site-mediated multiple-layer adsorption [23,32]. Moreover, the pseudo-first (3) and the pseudo-second (4) order of kinetic equations were also applied to analyze the kinetics of As(V) adsorption [23,33].

(3) $$In\left(Q_{e}-Q_{t}\right)=InQ_{e}-K_{1}t,$$

(4) $$\frac{1}{Q_{t}}=\frac{1}{k_{2}Q_{e}^{2}}+\frac{t}{Q_{e}},$$

where *Q_t_* (mg/g) is adsorption capacity at time *t* (h); and *K*_1_ (1/h) and *K*_2_ (g/mg·h) are adsorption rate constants of pseudo-first order and pseudo-second order, respectively. The adsorption kinetics of As(V) was well explained by the pseudo-second-order because the value of *R*^2^ for pseudo-second (*R*^2^ = 0.999) was higher than that of the pseudo-first (*R*^2^ = 0.984) (Table 2, Figure 5c,d). Furthermore, the value of *Q_exp_* (131.565 ± 3.783 mg/g) was almost identical to the value of *Q_cal_* (133.333 mg/g) in the pseudo-second-order model. Thus, it was predicted that the adsorption was achieved through the chemisorption process [34].

**Figure 6:**
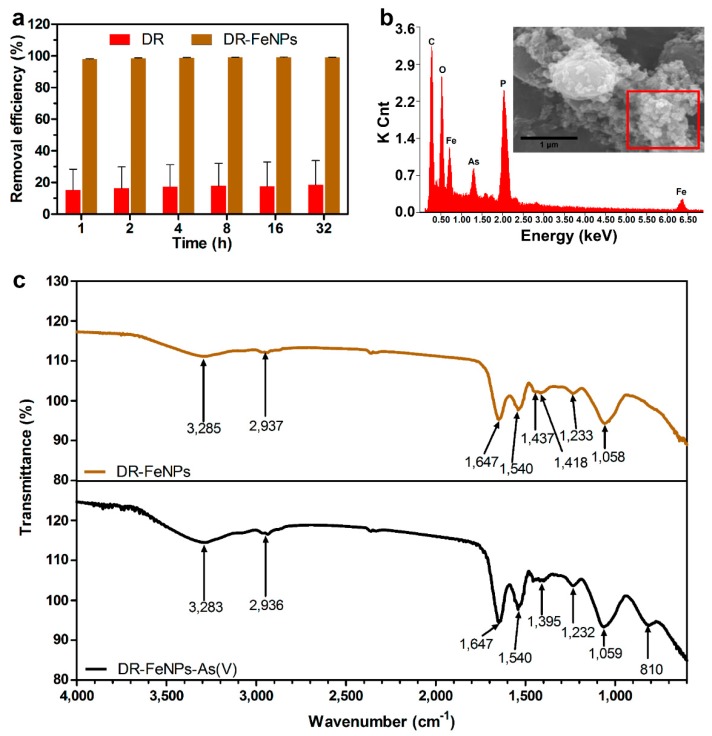
(**a**) Removal efficiency of *D. radiodurans* R1 (DR) and iron nanoparticle immobilized *D. radiodurans* R1 (DR-FeNPs). Experimental condition: initial pH = 3, biomass = 0.25 g/L, initial concentration of As(V) = 50 mg/L. All experiments were performed in triplicate; error bars denote standard deviation. (**b**) SEM-EDX analysis of DR-FeNPs with As(V) [DR-FeNPs-As(V)]. (**c**) FT-IR analysis of DR-FeNPs and DR-FeNPs-As(V).

Then, the removal capacity of DR-FeNPs for As(V) was further investigated under the optimized conditions. As can be seen in Figure 6a, rapid uptake kinetics and maximum removal efficiency (>98%) were successfully achieved within 1 h and the removal efficiency was maintained stable for up to 32 h (98.97%). The peaks corresponding As(V) in the EDX spectrum indicated that As(V) was selectively adsorbed by the DR-FeNPs (Figure 6b). Furthermore, the vibration of As–O at 810 cm^−1^ [23] analyzed by FT-IR analysis demonstrated that the FeNPs was selectively coupled with As(V) (Figure 6c). However, it was interesting to note that less than 17% of removal efficiency was also found in DR strain without FeNPs (Figure 6a). It seems to be because of the biological components such as terpenoids, amides, and aldehyde capable of chelating metal ions [35,36].

### 2.4. Effects of Competitive Anions on the Removal of As(V)

**Figure 7:**
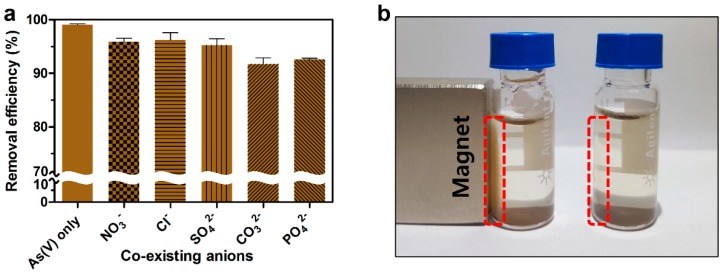
(**a**) Effect of co-existing anion on the removal efficiency. Experimental condition: initial pH = 3, biomass = 0.25 g/L, contact time = 16 h, initial concentration of As(V) = 50 mg/L. Initial concentration of co-existing anions: NO_3_^−^ = 10 mg/L, Cl^−^ = 200 mg/L, SO_4_^2−^ = 200 mg/L, CO_3_^2−^ = 50 mg/L, PO_4_^3−^ = 50 mg/L. All experiments were performed in triplicate; error bars denote standard deviation. (**b**) Recovery of DR-FeNPs-As(V) by magnet.

In most cases, the removal efficiency of As(V) is known to decrease dramatically under the presence of various competitive anions such as NO_3_^−^, Cl^−^, SO_4_^2−^, CO_3_^2−^, and PO_4_^3−^, which can block the active site of adsorbents resulting in the interference of coupling with As(V) [23,37,38,39]. Thus, the removal efficiency of DR-FeNPs for As(V) was evaluated in the presence of various competitive anions to simulate the practical application for the removal of As(V) contaminated natural underground water. Interestingly, the removal efficiency of DR-FeNPs for As(V) was scarcely influenced by competing anions. The removal efficiency was slightly decreased under the presence of competing anions, but more than 91% of removal efficiency was still maintained for 16 h (Figure 7a). It seems to be because of the cellular membrane-bound biological components that have the ability to chelate various co-existing ions and make a driving force that positively increases the removal efficiency and selectivity for As(V). Furthermore, owing to the magnetic property of FeNPs, the used DR-FeNPs could be easily recovered by employing magnets from aqueous solution within 24 h (Figure 7b). This is also one of the benefits of preventing secondary contamination by the used adsorbent, which is the obstacle to the bioremediation of environmental toxic pollutants.

## 3. Materials and Methods

### 3.1. Synthesis of DR-FeNPs

Iron chloride hexahydrate (FeCl_3_·6H_2_O) was purchased from JUNSEI Co. (Tokyo, Japan). *D. radiodurans* R1 strain (ATCC13939) was inoculated in TGY (0.5% tryptone, 0.1% glucose, and 0.3% yeast extract) and cultured at 30 °C with rotation at 200 rpm. The liquid medium was incubated until the sample reached an optical density of 1 at 600 nm (OD_600_). After cultivation, 6.25 mM FeCl_3_·6H_2_O was added to the cell and incubated for 24 h at 30 °C with 200 rpm. The cultures were centrifuged at 4000 rpm for 10 min at 4 °C, and the resulting pellets were washed two times with deionized water. The pellets were freeze-dried and used for further analysis.

### 3.2. Characterization of DR-FeNPs

The surface morphology and elemental analysis of *D. radiodurans* R1 and DR-FeNPs were observed by SEM-EDX (FEI Inspect F50, FEI, Hillsboro, OR, USA). For analysis by SEM-EDX, the samples were fixed with 2.5% glutaraldehyde solution and then dehydrated with 30%, 50%, 70%, 80%, 90%, 95%, and 100% ethanol. After dehydration with ethanol, the samples were further dried overnight. The size distribution of FeNPs was measured by DLS (Zetasizer Nano, Malvern Instruments, Worcestershire, UK). To isolate FeNPs from DR-FeNPs, the DR-FeNPs were disrupted by using bead beater (FastPrep-24 Instrument, MP Biomedical, Santa Ana, CA, USA) at 6000 rpm for 1 min. Then, disrupted samples were filtered by 0.45 μm cellulose acetate membrane (Hyundai Micro, Seoul, Korea). Zeta potential of DR-FeNPs was measured with a zeta-potential and particle size analyzer (ELSZ-1000, OTSUKA ELECTRONICS, Osaka, Japan). FT-IR spectra of the materials were recorded with KBr disks using the FT-IR spectrometer (Thermo Scientific NICOLET iS10, Thermo Fisher Scientific, Waltham, MA, USA) in the range of 4000–600 cm^−1^. The crystallinity of DR-FeNPs was monitored by XRD (D max-2500 PC, RIGAKU, Tokyo, Japan) from 2*θ* = 10°–70°. The surface states of DR-FeNPs were characterized by XPS (K-alpha plus, Thermo Fisher Scientific, Waltham, MA, USA) using an Al Kα (*hv* = 1486.7 eV) micro-focused monochromator. All spectra were calibrated using the adventitious C 1s peak with a fixed value of 284.8 eV.

### 3.3. Optimization of As(V) Adsorption Using DR-FeNPs

Disodium hydrogen arsenate heptahydrate (Na_2_HAsO_4_·7H_2_O) was purchased from Wako Co. (Tokyo, Japan). A stock solution of 1000 mg/L was obtained by dissolving Na_2_HAsO_4_·7H_2_O in deionized water. The effect of several parameters such as pH, contact time, amount of biomass, and initial concentration of As(V) on biosorption efficiency was evaluated. To determine optimum pH value, biosorption experiments were performed in an Erlenmeyer flask containing 10 mL solution for 24 h at 30 °C with 200 rpm. The initial pH of the solution was adjusted using 0.1 M HCl and 0.1 M NaOH solution. The amount of biomass and initial concentration of As(V) were fixed at 0.2 g/L and 50 mg/L, respectively.

The effect of contact time on biosorption efficiency in the range of 1–32 h was conducted in an Erlenmeyer flask containing 10 mL of As(V) solution with 50 mg/L biomass. The other parameters including pH, agitation, biomass, and temperature were kept constant at 3, 200 rpm, 0.25 g/L, and 30 °C, respectively.

Further experiments were carried out to investigate the effect of biomass and initial concentration of As(V) on biosorption efficiency in ranges of 0.25–5 g/L and 50–500 mg/L, respectively. pH, agitation, and temperature were kept constant at 3, 200 rpm, and 30 °C, respectively.

The samples were harvested at 4000 rpm for 10 min. The supernatants were filtered through a 0.45 μm cellulose acetate filter. The residual As(V) in the supernatant was measured by inductively coupled plasma optical emission spectroscopy (ICP-OES, iCAP 7000 series, Thermo Scientific, Waltham, MA, USA). All biosorption assays were performed in triplicate and the mean values were calculated.

### 3.4. Remediation Procedure of As(V) Using DR-FeNPs

The amount of 2.5 g/L of *D. radiodurans* R1 and DR-FeNPs was added to pH 3.0 solution containing 50 mg/L of As(V). The mixtures were shaken at 30 °C with 200 rpm. At each time point, 1, 2, 4, 8, 16, and 32 h samples were withdrawn from the solution. The supernatants were filtered through a 0.45 μm pore size cellulose acetate filter. The residual As(V) in the supernatant was measured by ICP-OES (inductively coupled plasma-optical emission spectrometry).

The removal efficiency (%) was defined by the following equation to assess the adsorption capacity of DR-FeNPs for toward As(V):

Removal efficiency (%) = (C_0_ − C_e_)/C_0_ × 100, (5)

where C_0_ and C_e_ represent the initial and equilibrium concentrations of As(V), respectively. DR-FeNPs with As(V) [DR-FeNPs-As(V)] was analyzed by SEM-EDX. FT-IR spectra of DR-FeNPs and DR-FeNPs-As(V) were recorded with KBr disks using a FT-IR spectrometer in the range of 4000–600 cm^−1^.

### 3.5. Analysis of the Effect of Co-Existing Ion on As(V) Adsorption

Sodium phosphate tribasic dodecahydrate (Na_3_PO_4_·12H_2_O), sodium sulfate anhydrase (Na_2_SO_4)_, sodium nitrate (NaNO_3_), and sodium carbonate anhydrous (Na_2_CO_3_) were purchased from DAEJUN Co. (Gyeong gi, Siheung, Korea). To study the effect of co-existing anions on the removal of As(V), 10 mg/L NO_3_^−^, 200 mg/L Cl^−^, 200 mg/L SO_4_^2−^, 50 mg/L CO_3_^2−^, and 50 mg/L PO_4_^3−^ were treated under 2.5 g/L of DR-FeNPs and 50 mg/L of As(V) [40]. The pH was adjusted to 3 with the addition of 0.1 M HCl solution. Suspensions were shaken for 16 h at 30 °C with rotation at 200 rpm. The supernatants were filtered through a 0.45 μm cellulose acetate filter. The residual As(V) in the supernatant was measured by ICP-OES.

## 4. Conclusions

We developed a new bioremediation platform technology using a magnetic iron nanoparticle immobilized *D. radiodurans* R1 strain (DR-FeNPs) capable of removing toxic arsenic species [As(V)]. This was achieved in vivo, by the synthesis of magnetic iron nanoparticles in the presence of strong reducing biomolecules in a living *D. radiodurans* R1 (DR) strain. The As(V) removal method using DR-FeNPs developed here showed an adsorption capacity of 131.6 mg/g, which has the highest removal efficiency among the methods using iron nanomaterials (Table 3). The bioremediation process using DR-FeNPs has the following advantages over the conventional physicochemical methods: (1) various types of nanocomposite can be synthesized by controlling the culture condition without seasonal effects; (2) key drawbacks of physicochemical methods such as time-consumption, high-cost, and labor-intensiveness can be overcome; and (3) secondary contamination resulting from the used DR-FeNPs can be prevented by employing magnets, as can be seen in Figure 7b. Also, if desired, systems metabolic engineering can be applied to design customized microorganisms capable of removing a variety of toxic substances. Furthermore, our bioremediation method based on a living DR strain is not limited to removing As(V), but can be extended to the removal of other toxic substances by in vivo synthesis of a variety of nanomaterials.

## Figures and Tables

**Table 1:** Langmuir and Freundlich model parameters. DR-FeNPs, magnetic iron nanoparticle-immobilized *D. radiodurans* R1 strain.

Biomass	Langmuir Isotherm			Freundlich Isotherm		
	*Q*_0_ (mg/g)	*K_L_* (L/mg)	*R* ^2^	*K_F_* (L/g)	*n*	*R* ^2^
DR-FeNPs	172.414	0.176	0.870	25.146	1.596	0.961

**Table 2:** Pseudo-first order and pseudo-second-order kinetic models.

**Pseudo-First Order**	*Q_exp_* (mg/g)	*K*_1_ (1/h)	*Q_cal_* (mg/g)	*R* ^2^
	131.565 ± 3.783	0.197	32.089	0.984
**Pseudo-Second Order**	*Q_exp_* (mg/g)	*K*_2_ (g/mg·h)	*Q_cal_* (mg/g)	*R* ^2^
	131.565 ± 3.783	0.018	133.333	0.999

**Table 3:** Comparison of iron nanomaterial adsorbent for As(V) removal.

Adsorbent	Preparation Method	Adsorption Capacity	pH	Ref
Magnetic nanoparticles coated zeolite	Ultra sonication	95.6%	2.5	[41]
Fe-hydrotalcite supported mangetic nanopartilces (M-FeHT)	Co-precipitation	104.8 μg/g	9	[42]
Green nano iron particle (GnIP)	Mint leaves extract	94.47 mg/g	-	[43]
Ascrobic acid coated Fe_3_O_4_ nanoaprticle	Hydorthermal (180 °C)	16.56 mg/g	7	[44]

## Abbreviations

DR	*D. radiodurans* R1
DR-FeNPs	Iron nanoparticle immobilized *D. radiodurans* R1
DR-FeNPs-As(V)	DR-FeNPs adsorbing As(V)
*Q_e_*	Adsorption capacity at equilibrium
*Q_t_*	Adsorption capacity at any time
*Q*_0_	Maximum adsorption capacity
*Ce*	Concentration of metal ions in aqueous solution at equilibrium
*K_L_*	Langmuir adsorption constant
*K_F_*	Freundlich adsorption constant
1/*n*	Adsorption intensity
*Q_exp_*	Adsorption capacity at equilibrium (experimental value)
*Q_cal_*	Adsorption capacity at equilibrium (calculation value)
*K*_1_	Adsorption rate constant of the pseudo-first model
*K*_2_	Adsorption rate constant of the pseudo-second model
XRD	X-ray diffraction
XPS	X-ray photoelectron spectroscopy
SEM-EDX	Scanning electron microscopy-energy dispersive X-ray spectroscopy
